# Endomucin selectively regulates vascular endothelial growth factor receptor-2 endocytosis through its interaction with AP2

**DOI:** 10.1186/s12964-024-01606-w

**Published:** 2024-04-11

**Authors:** Issahy Cano, Melissa Wild, Urvi Gupta, Suman Chaudhary, Yin Shan Eric Ng, Magali Saint-Geniez, Patricia A. D’Amore, Zhengping Hu

**Affiliations:** 1 Schepens Eye Research Institute of Massachusetts Eye and Ear, Boston, MA USA; 2 Department of Ophthalmology, Harvard Medical School, Boston, MA USA; 3 Department of Pathology, Harvard Medical School, Boston, MA USA; 4 Case Western Reserve University School of Medicine, Cleveland, OH USA; 5 Present affiliation: Department of Molecular Medicine, Cornell University, Ithaca, NY USA; 6 Present Affiliation: EyeBiotech, London, UK; 7 Present affiliation: Novartis Institutes for Biomedical Research, Cambridge, MA USA

**Keywords:** Endothelium, Endocytosis, Adaptor protein, Fibroblast growth factor receptor 1 (FGFR1), Glycocalyx, Vascular endothelial growth factor receptor 1 (VEGFR1)

## Abstract

The endothelial glycocalyx, located at the luminal surface of the endothelium, plays an important role in the regulation of leukocyte adhesion, vascular permeability, and vascular homeostasis. Endomucin (EMCN), a component of the endothelial glycocalyx, is a mucin-like transmembrane glycoprotein selectively expressed by venous and capillary endothelium. We have previously shown that knockdown of EMCN impairs retinal vascular development in vivo and vascular endothelial growth factor 165 isoform (VEGF165)-induced cell migration, proliferation, and tube formation by human retinal endothelial cells in vitro and that EMCN is essential for VEGF165-stimulated clathrin-mediated endocytosis and signaling of VEGF receptor 2 (VEGFR2). Clathrin-mediated endocytosis is an essential step in receptor signaling and is of paramount importance for a number of receptors for growth factors involved in angiogenesis. In this study, we further investigated the molecular mechanism underlying EMCN’s involvement in the regulation of VEGF-induced endocytosis. In addition, we examined the specificity of EMCN's role in angiogenesis-related cell surface receptor tyrosine kinase endocytosis and signaling. We identified that EMCN interacts with AP2 complex, which is essential for clathrin-mediated endocytosis. Lack of EMCN did not affect clathrin recruitment to the AP2 complex following VEGF stimulation, but it is necessary for the interaction between VEGFR2 and the AP2 complex during endocytosis. EMCN does not inhibit VEGFR1 and FGFR1 internalization or their downstream activities since EMCN interacts with VEGFR2 but not VEGFR1 or FGFR1. Additionally, EMCN also regulates VEGF121-induced VEGFR2 phosphorylation and internalization.

**Graphical Abstract:**

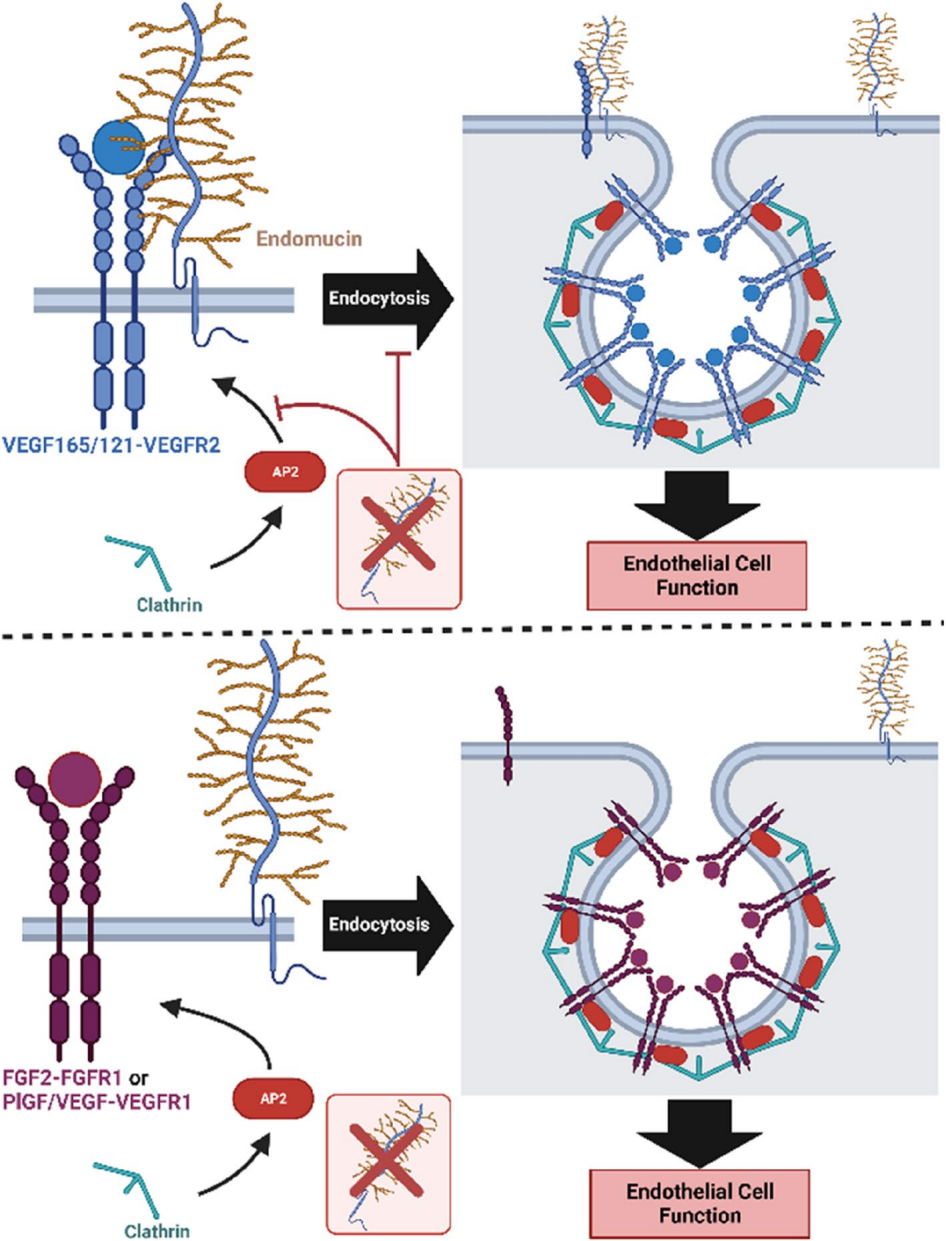

**Supplementary Information:**

The online version contains supplementary material available at 10.1186/s12964-024-01606-w.

## Introduction

Angiogenesis, the process by which new capillaries develop from pre-existing vessels, is necessary for a wide range of homeostatic and pathological processes that range from embryonic development to wound healing and tumor growth [50, 56, 58]. During angiogenesis, endothelial cells (ECs) proliferate, migrate, and form tube-like structures [2, 6]. New vessel formation is driven by a multitude of growth factors that bind to corresponding receptors expressed on the endothelial surface [7, 17]. One of the most well-studied angiogenic growth factors is vascular endothelial growth factor A (VEGF-A). VEGF-A, a member of the VEGF/PDGF superfamily, is produced by alternative mRNA splicing into a number of isoforms, including VEGF121, 145, 148, 165, 183, 189, and 206 in humans [6]. VEGF165, a heparin-binding VEGF-A isoform, is the best-studied isoform and has been shown to be a major stimulator of angiogenesis. VEGF-A mediates its effects via binding to VEGF receptor 2 (VEGFR2), inducing receptor tyrosine kinase phosphorylation and activating downstream signaling cascades and transcriptional changes that drive the various steps of angiogenesis by ECs [16, 17, 39, 54]. VEGFR1, an additional VEGF-A binding receptor, has a greater affinity for VEGF-A than VEGFR2. It is thought to primarily act as a decoy receptor for VEGF-A at the endothelial surface [5, 18]. In addition to VEGF-A, placental growth factor (PlGF), a member of the VEGF family of ligands, also binds to VEGFR1 [10]. Interestingly, PlGF indirectly modulates VEGF-A’s availability to interact with VEGFR2 by competing for VEGFR1 binding [35]. Different isoforms of VEGF-A promote varying rates of VEGFR2 internalization. Compared to VEGF165, and despite inducing phosphorylation of VEGFR2 at the Y1175 site, VEGF121 promotes a slower rate of VEGFR2 internalization [15]. Basic fibroblast growth factor 2 (FGF2), a member of the extensive family of secreted FGF angiogenic growth factors, signals through tyrosine kinase receptors expressed on the EC surface. Similar to VEGF165, FGF2 induces EC migration, proliferation, and tube formation in vitro [3] and angiogenesis in vivo [20].

Upon ligand binding, VEGFR2 is internalized primarily by clathrin-mediated endocytosis (CME) along with alternative endocytosis pathways [1, 52]. The amplitude, duration, and propagation of receptor signaling are strongly influenced by their endocytosis [21, 53], and in some cases, receptors like VEGFR2 continue to signal intracellularly [31, 32]. Following ligand-induced endocytosis, depending on ligand sensitization and membrane receptor levels, VEGFR2 may be ubiquitinated and degraded, or recycled to the cell-surface [4, 13]. CME is characterized by the formation of clathrin-coated pits at the cell surface membrane, which invaginate forming intracellular vesicles that contain cell surface components and specific extracellular cargo [13, 60]. Since clathrin lacks the ability to bind directly to the lipids or proteins present in the plasma membrane, adaptor proteins play a critical role in orchestrating the formation of clathrin-coated vesicles by establishing interactions between clathrin and the cargo molecules embedded in the membrane [37]. One of the primary adaptor proteins with which clathrin associates at the plasma membrane is adaptor protein 2 (AP2). In addition to its interaction with clathrin, AP2 also associates with many binding partners, including internalization receptors as well as other adaptor proteins that play a role in facilitating the process of endocytosis [55]. Much like VEGFR2, VEGFR1 and FGF receptor 1 (FGFR1) are known to endocytose predominantly via CME upon ligand activation [24, 25, 40].

The endothelial glycocalyx layer is comprised of glycoproteins, glycosaminoglycans, and proteoglycans. This meshwork of molecules located at the apical cell surface, regulates leukocyte adhesion, vascular homeostasis, and endothelial function [11, 12, 46]. Endomucin (EMCN), a mucin-like transmembrane glycoprotein and component of the endothelial glycocalyx, is selectively expressed in venous and capillary endothelium [34]. Previously our lab has established that EMCN is involved in retinal vascular development in vivo and that its absence impairs EC proliferation, migration, and tube-formation [44]. In addition, we have reported that EMCN plays a critical role in VEGF165-induced CME of VEGFR2 and subsequent signaling [33]. The underlying molecular mechanism by which EMCN controls the endocytosis of VEGFR2 is not yet fully understood. In this study, we further explored the molecular mechanism of EMCN's role in modulating VGFR2 endocytosis by identifying its potential protein binding partners, defining the role of EMCN in VEGFR2 activation induced by the non-heparin binding VEGF121 isoform, and determining the specificity of EMCN in modulating other endothelial receptor tyrosine kinases involved in angiogenesis.

## Results

### Mass spectrometry identifies AP2 as an EMCN binding partner

**Fig. 1:**
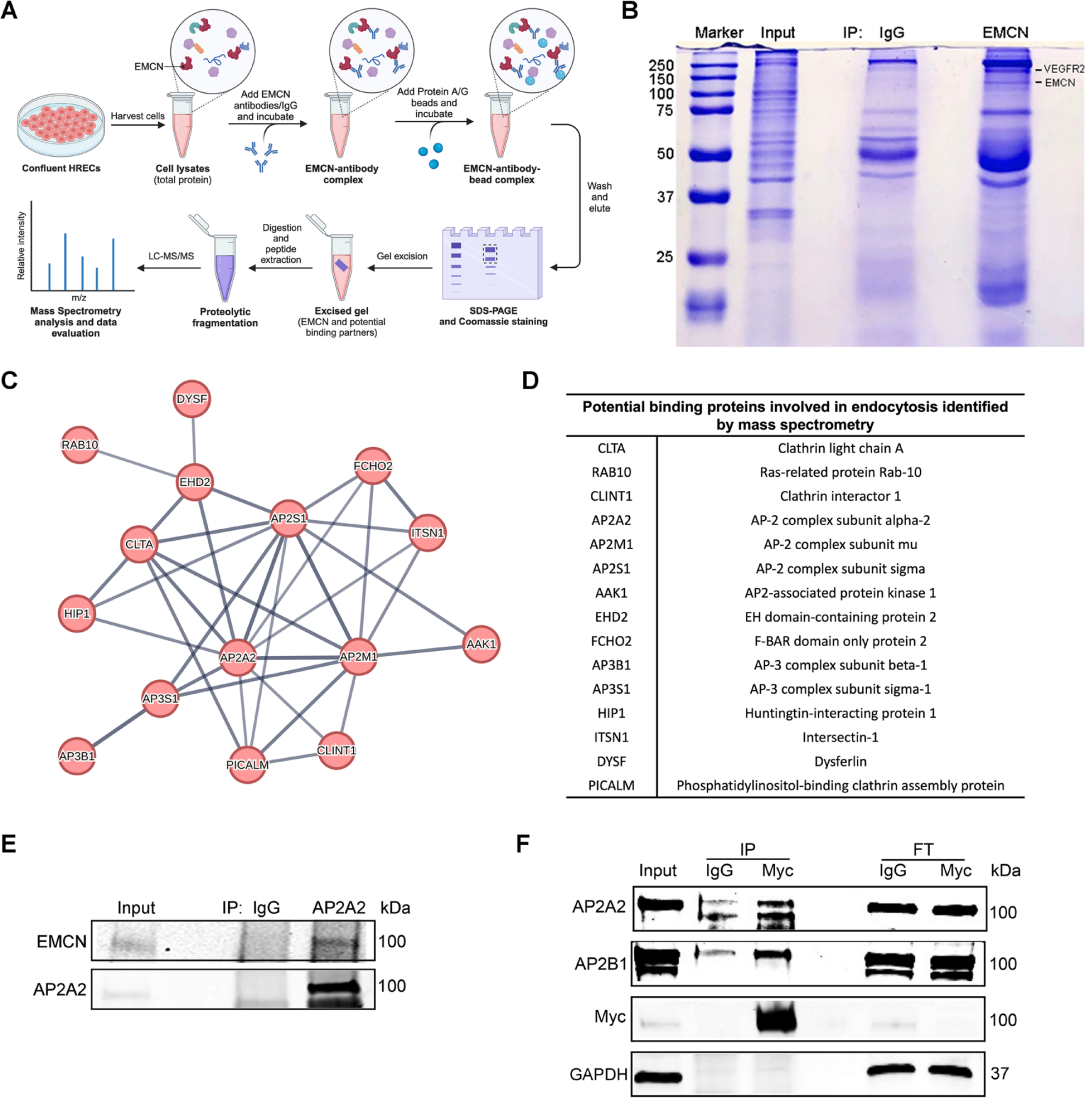
Potential binding proteins of EMCN involved in endocytosis identified through mass spectrometry. **A** Diagram illustrating the workflow of EMCN immunoprecipitation in HRECs lysate and subsequent identification of potential EMCN-binding proteins using mass spectrometry. **B** Coomassie blue stained SDS-PAGE gel showing the separation of EMCN binding proteins, which were then excised for mass spectrometric analysis. **C** Protein clusters involved in endocytosis among EMCN-binding proteins identified by mass spectrometry and analyzed using the STRING database. **D** List of endocytosis related EMCN binding proteins identified in mass spectrometry. **E** Validation of the interaction between EMCN and AP2A2 by immunoprecipitation on Western blot. **F** HRECs overexpressing myc-tagged EMCN were lysed and both AP2 α and β subunits co-immunoprecipitated with EMCN. FT: flowthrough. *n* = 3

We have previously shown an essential role for EMCN in mediating CME of VEGFR2 [33], and specifically that the EMCN extracellular domain is involved in the EMCN-VEGFR2 interaction [27]. To further explore the molecular mechanism of EMCN in CME of VEGFR2, we performed co-immunoprecipitation of EMCN in human retinal endothelial cells (HRECs) followed by mass spectrometry to identify the potential protein binding partners of EMCN (Fig. 1A). EMCN or IgG control immunoprecipitated products were separated in SDS-PAGE gel and visualized by Coomassie blue staining for mass spectrometry as shown in Fig. 1B. Annotated EMCN binding proteins (416 in total) from mass spectrometry were analyzed using the STRING database for functional clustering, and proteins involved in endocytosis appear in one of the top functional clusters (Supplemental Fig. 1A and B). The cluster of EMCN-binding proteins involved in endocytosis is visualized in Fig. 1C and the protein names are listed in Fig. 1D. Several subunits or proteins involved in the AP2 complex of the CME pathway were found on the list, including AP2A2, AP2M1, AP2S1, and AAK1. The interaction between EMCN and the AP2A2 subunit was validated using immunoprecipitation as shown in Fig. 1E. We further examined the interaction between EMCN and AP2β, the other major subunit of AP2 complex. Immunoprecipitation of EMCN (Myc-tagged EMCN with anti-Myc antibody) from HRECs revealed that EMCN interacts with both α and β subunit of AP2 adaptor complex (*n* = 3; Fig. 1F).

### AP2 is essential for the CME of VEGFR2

**Fig. 2:**
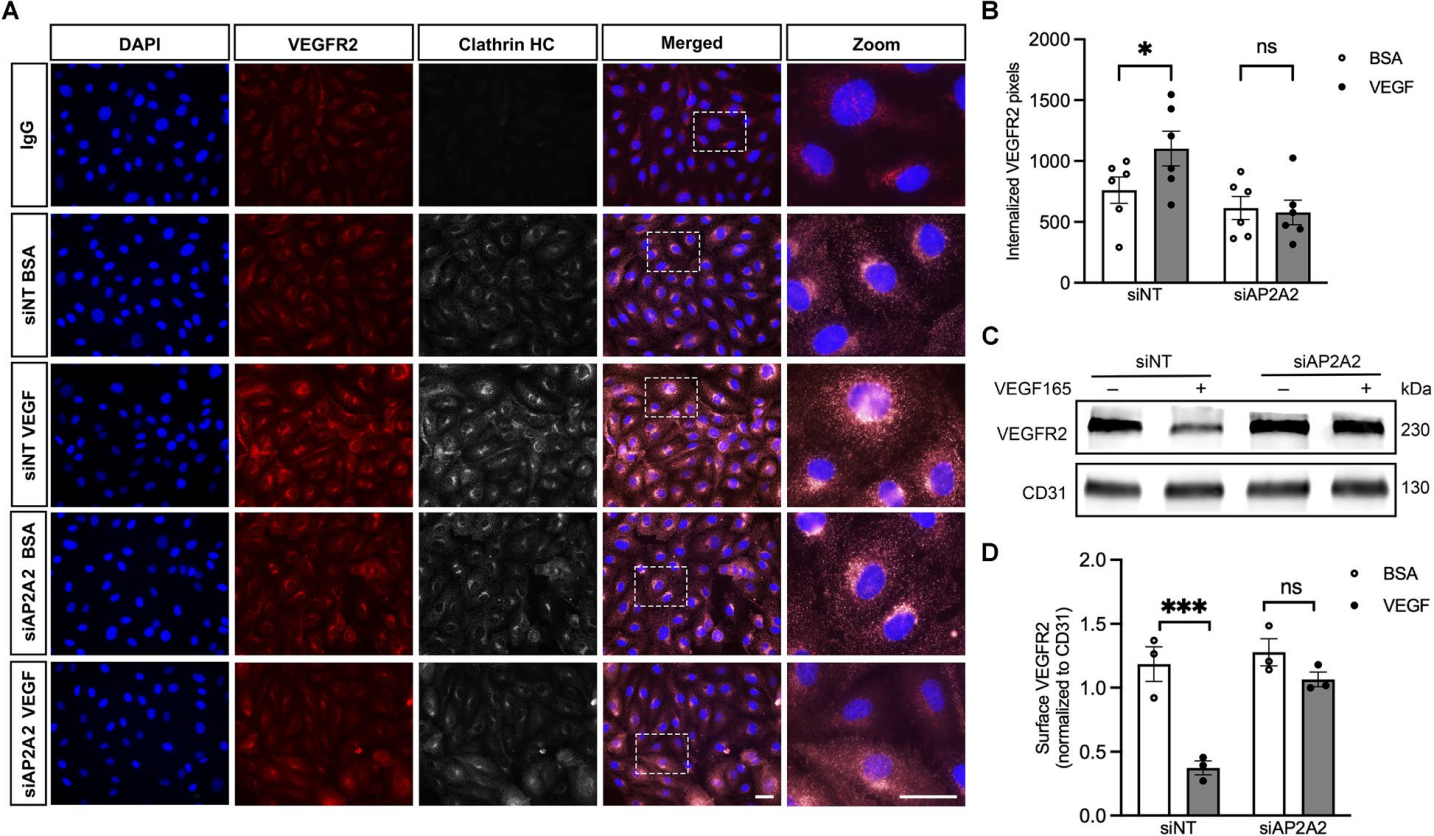
AP2 adaptor complex is essential for VEGFR2 CME following VEGF165 stimulation. **A** Internalized VEGFR2 following VEGF stimulation or BSA control was visualized by intracellular fluorescence intensity (red). Clathrin HC (heavy chain) (white) was co-visualized in each condition. In the absence of AP2A2 subunit, VEGF-induced VEGFR2 internalization (red) was reduced after 30 min. Bar = 20 µm **B** VEGFR2 internalization was quantified by relative fluorescence intensity and normalized to the total cell number per viewing field. Student-t test was used for statistical analysis. *P < 0.05, *n* = 6. **C** HRECs incubated in serum-free media were stimulated with VEGF165 (10 ng/ml) for 30 min with and without AP2A2 subunit knockdown, and cell surface VEGFR2 levels were analyzed by western blot. **D** Quantification of cell surface VEGFR2 band intensity normalized to CD31. One-way ANOVA was used for statistical analysis. ***P < 0.001, *n* = 3

The AP2 complex is a major adaptor and plays a crucial role in CME [9, 30]. We further investigated the role of the AP2A2 subunit in VEGFR2 endocytosis by tracking Internalized VEGFR2 in HRECs where we had previously shown enhanced VEGFR2 signaling by VEGF treatment via CME. AP2A2 subunit knocked down in HRECs resulted in significantly less internalized VEGFR2 compared to non-targeting siRNA (siNT) control (Fig. 2A) upon VEGF stimulation. Quantification of internalized VEGFR2 confirmed a significant increase in internalized VEGFR2 was observed following VEGF stimulation compared to BSA control (1103.02 ± 143.1 vs. 760.63 ± 108.82 pixels, *p* < 0.05, *n* = 6) (Fig. 2B). AP2A2 subunit knockdown effectively blocked VEGF-stimulated VEGFR2 internalization (578.40 ± 100.53 vs. 614.63 ± 94.45 pixels, *p* > 0.05, *n* = 6. Cell surface biotinylation and quantification of the VEGFR2 band intensity (Fig. 2C and D) confirmed that the knockdown of the AP2A2 subunit significantly blocked the VEGF165-induced internalization of VEGFR2, compared to the control. Confirmation of EMCN knockdown at both mRNA level and protein level is shown in Supplemental Fig. 2.

### EMCN is required for VEGFR2 interaction with AP2 complex and clathrin recruitment

**Fig. 3:**
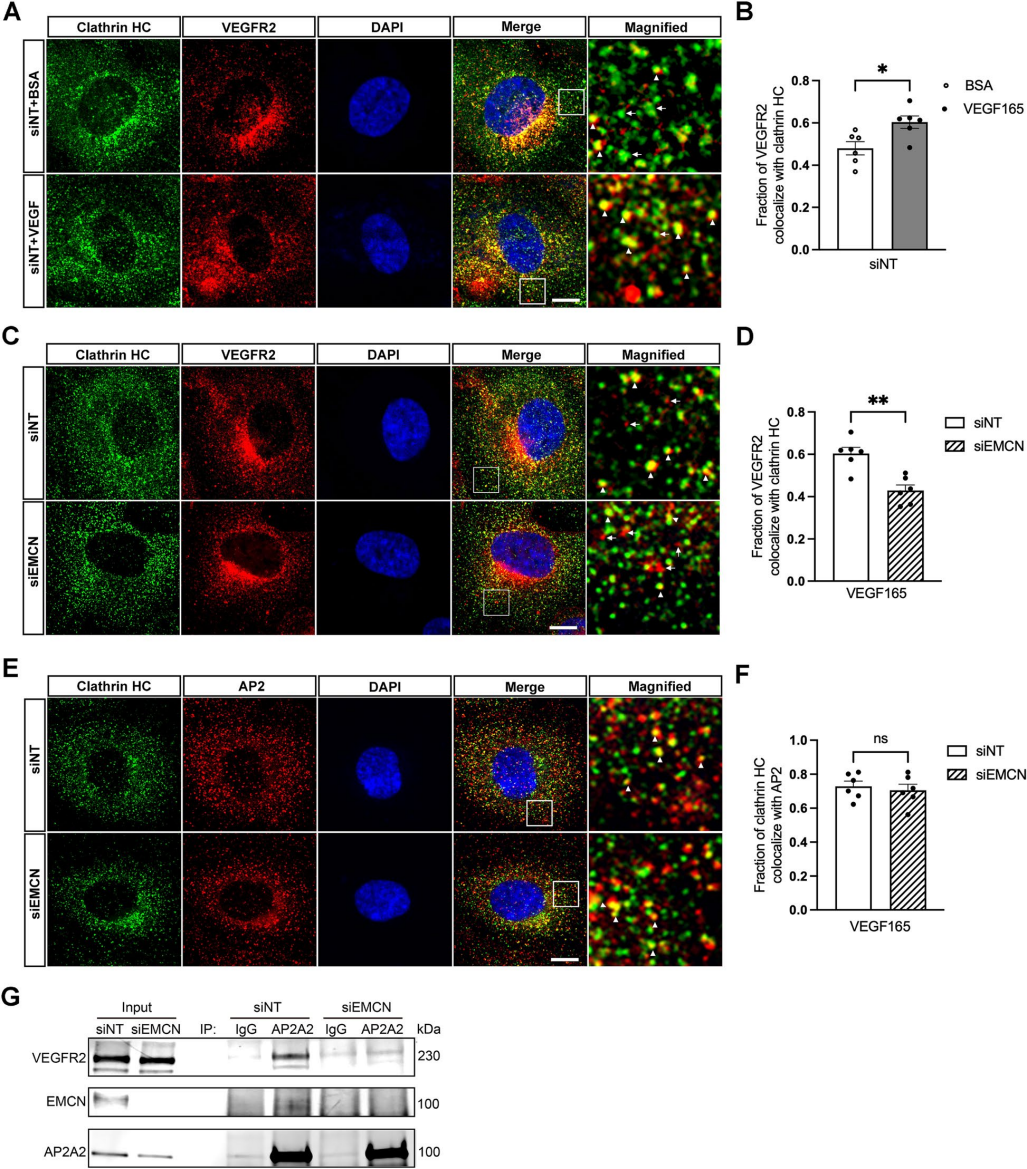
Essential role of EMCN in facilitating VEGFR2 and AP2 interaction and clathrin recruitment. **A** Colocalization of clathrin HC (heavy chain) (green) and VEGFR2 (red) were visualized in control HRECs with or without VEGF165 (10 ng/ml). Examples of colocalization of VEGFR2 and clathrin HC (white arrowhead), and clathrin (white arrow) were shown in magnified view. Bar = 10 µm **B** Fraction of VEGFR2 that colocalized with clathrin was quantified by Image J CoJAP plugin. Student t-test was used for the comparison. *P < 0.05, *n* = 3. **C** Colocalization of clathrin HC (heavy chain) (green) and VEGFR2 (red) were visualized in siNT or siEMCN HRECs with VEGF165 (10 ng/ml) stimulation. Examples of colocalization of VEGFR2 and clathrin HC (white arrowheads), and VEGFR2 (white arrow) were shown in magnified view. Bar = 10 µm **D** Fraction of VEGFR2 that colocalized with clathrin in siNT and siEMCN HRECs was quantified. Student t-test was used for the comparison. **P < 0.01, *n* = 3. **E** Colocalization of clathrin HC (heavy chain) (green) and AP2 (red) were visualized in control in siNT and siEMCN HRECs with VEGF(10 ng/ml) stimulation. Examples of colocalization of clathrin HC and AP2 (white arrowhead) were shown in magnified view. Bar = 10 µm (**F**) Fraction of clathrin that colocalized with AP2 in siNT and siEMCN HRECs with VEGF stimulation were quantified. Student t-test was used for the comparison. *P* > 0.05, *n* = 3. **G** EMCN is required for interaction between VEGFR2 and AP2A2. HRECs were lysed and VEGFR2 that co-immunoprecipitated with AP2A2 in the presence and absence of EMCN was observed. *n* = 3

To investigate the mechanism by which EMCN is involved in VEGFR2 CME, we examined the colocalization of VEGFR2 with clathrin. Upon VEGF stimulation of control HRECs, the fraction of VEGFR2 colocalized with clathrin significantly increased (Fig. 3 A and B). Although no significant differences were detected between siNT and siEMCN under the non-stimulated condition, the fraction of VEGFR2 that colocalized with clathrin was lower in the EMCN knockdown HRECs when compared to the control after VEGF stimulation (Fig. 3C and D). We then explored whether EMCN plays a role in the recruitment of clathrin by the AP2 complex. We quantified the co-localization of clathrin with the AP2A2 subunit following VEGF stimulation. There was no significant difference in the fraction of clathrin co-localized with AP2 between siEMCN-treated HRECs and siNT -ontrol cells (Fig. 3E and F). Building upon previous findings demonstrating EMCN's interaction with VEGFR2 on the cell surface [33], we investigated the interaction between VEGFR2 and the AP2A2 subunit in the presence or absence of EMCN through immunoprecipitation (Fig. 3G). In control cells treated with siNT, we observed an interaction between VEGFR2, AP2A2, and EMCN’ this interaction was not observed in cells with EMCN knockdown (*n* = 3).

### EMCN does not modulate VEGFR1-mediated HRECs migration or VEGFR1 internalization

**Fig. 4:**
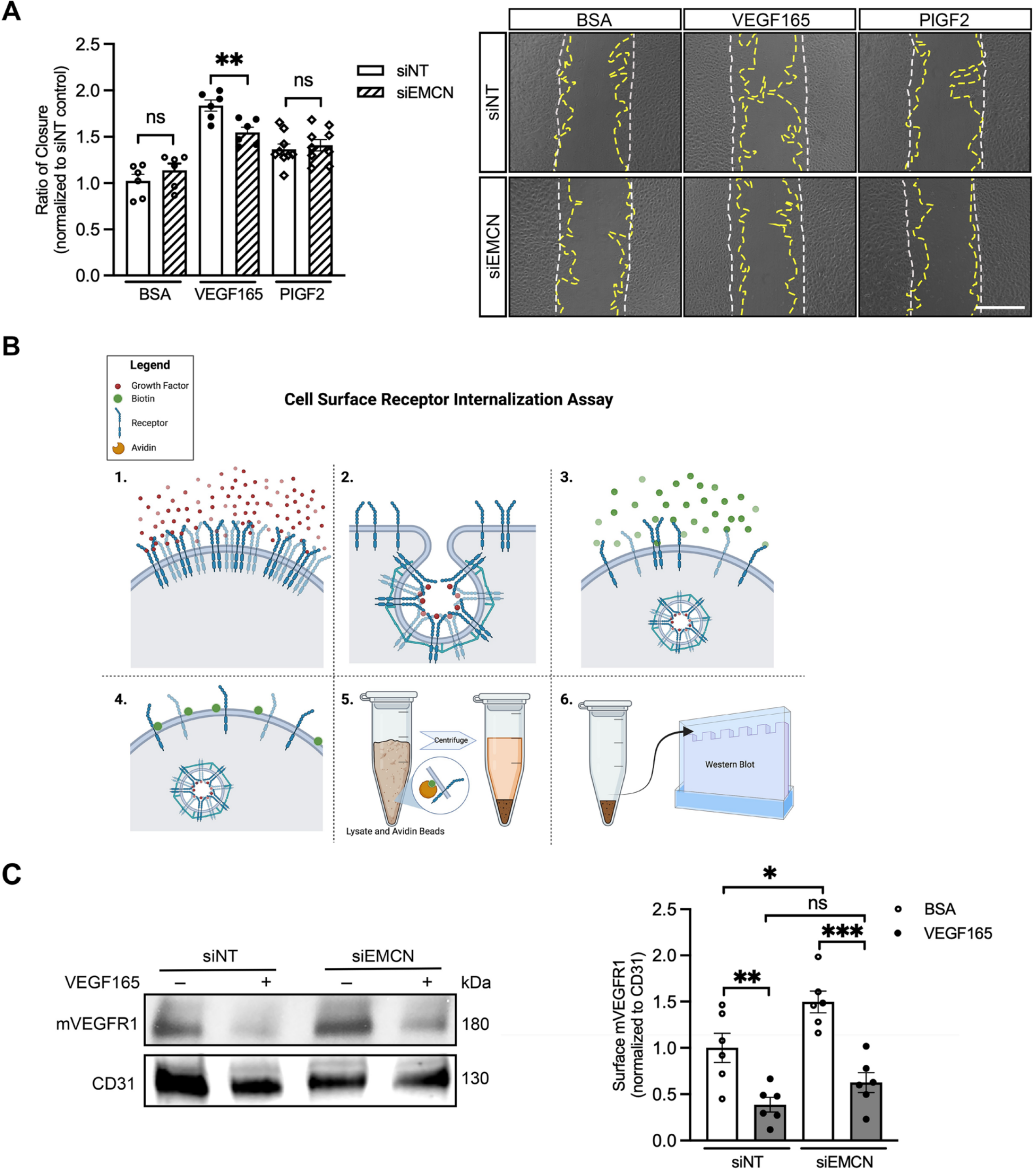
EMCN does not modulate VEGF165 or PIGF-induced endothelial migration or VEGFR1 internalization. **A** EMCN knockdown did not affect PlGF-2-induced HREC migration. HRECs were transfected with either siNT or siEMCN, mechanically scratched, stimulated with PlGF-2 (10 ng/ml) or VEGF165 (10 ng/ml), and the resulting cell migration was quantified by image analysis (left). **P < 0.01, *n* = 6 or 9. Representative images of each group at time zero (white dashed line) and 15 h time (yellow dashed line) points (right). The scale bar represents 500 µm. **B** Illustration of the cell surface receptor internalization assay. Growth factors bind to its cell surface receptors and induce receptor internalization. Cell surface proteins are biotinylated, the cell surface fraction is separated using avidin resin, and western blot analysis were used to analyze the fraction of receptors remaining at the cell surface. **C** HRECs incubated in serum-free media were stimulated with VEGF165 (10 ng/ml) for 30 min with and without EMCN knockdown, and cell surface membrane-bound VEGFR1 (mVEGFR1) levels were analyzed by western blot analysis. *P < 0.05, **P < 0.01, ***P < 0.001, *n* = 6. One-way ANOVA was used for statistical analysis

We next investigated whether the role of EMCN in receptor internalization was specific to VEGFR2. We therefore examined the effect of EMCN knockdown on the internalization of VEGFR1. Placental growth factor (PIGF), a VEGFR1 specific ligand, was used to investigate the effects of EMCN knockdown on VEGFR1-mediated HREC migration. HRECs stimulated with 10 ng/ml of PlGF-2 induced a significant migratory response (1.00 ± 0.0706 vs. 1.365 ± 0.0574 *P* < 0.01, *n* = 6 or 9), and EMCN knockdown had no significant effect on PlGF-2-induced migration of HRECs (1.365 ± 0.0841 vs. 1.409 ± 0.0434, *P* > 0.05, *n* = 6 or 9), whereas VEGF165-induced migration was significantly inhibited (1.837 ± 0.0826 vs. 1.548 ± 0.0289, *P* < 0.01, *n* = 6) (Fig. 4A).

To determine whether EMCN plays a role in VEGF165-induced VEGFR1 internalization, the level of cell surface VEGFR1 was quantified using biotinylation following VEGF165 stimulation (10 ng/ml) in the presence and absence of EMCN. Cell surface proteins were conjugated with biotin and extracted using avidin beads, and VEGFR1 protein was analyzed by western blot (Fig. 4B). The results showed that EMCN depletion did not affect the ability of VEGF165 to induce VEGFR1 internalization after 30 min compared to control (0.625 ± 0.239 vs. 0.386 0.134 ± , *P* > 0.05, *n* = 6) (Fig. 4C). The observation of increased baseline cell surface VEGFR1 levels upon knockdown of EMCN in HRECs is similar to what was observed with VEGFR2 levels following EMCN knockdown [33].

### EMCN is not required for FGF2-induced cell migration or FGFR1 internalization

**Fig. 5:**
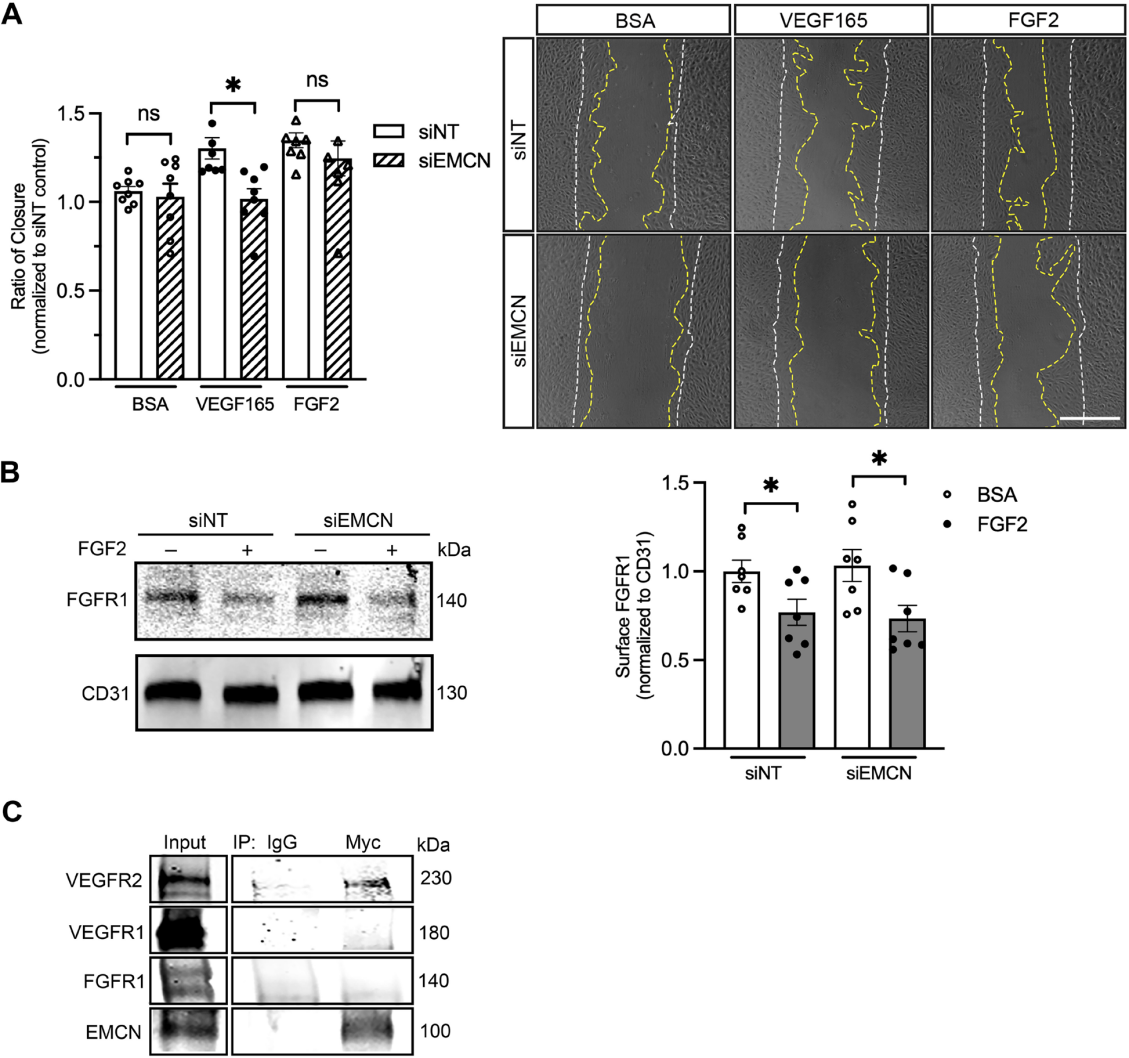
EMCN is not required for FGF2-induced HREC cell migration or FGFR1 internalization. **A** EMCN knockdown did not affect FGF2-induced HREC migration. HRECs were transfected with either siNT or siEMCN, incubated in serum-free media for 8 h, mechanically scratched, and stimulated with FGF2 (10 ng/ml) or VEGF165 (10 ng/ml). Quantification of cell migration for all the treatment groups based on image analysis (left). Student t-test was used for comparisons within groups. *P < 0.05, *n* = 8. Representative images of each group at time zero (white dashed line) and 15 h time (yellow dashed line) points (right). The scale bar represents 500 µm. **B** EMCN knockdown did not affect FGF2-induced FGFR1 internalization in HREC. Serum-starved HRECs were stimulated with FGF2 for 45 min, and then the cell surface proteins were isolated and visualized by western blot. Quantification of FGFR1 at the cell surface from all treatment groups by western blot analysis (left). Student-t test was used for statistical analysis. *P < 0.05, *n* = 7. A representative western blot for all treatment groups (right). **C** EMCN does not interact with VEGFR1 or FGFR1 in HRECs. HRECs overexpressing myc-tagged EMCN were lysed, and cell surface receptors that co-immunoprecipitated with EMCN were observed. *n* = 3. Note that the IgG and Myc groups were overexposed together for the better detection of the different receptors because of the low protein levels, while the input groups were kept at a lower exposure

Given the similarities between VEGFR2 and FGFR1 in ligand-driven endocytosis and downstream angiogenesis signaling, as well as the fact that both are expressed in vascular endothelial cells, we investigated whether EMCN plays a role in FGF2-induced HREC migration and FGFR1 internalization. FGF2 induced HREC migration (1.00 ± 0.0228 vs. 1.248 ± 0.0354, *P* < 0.001, *n* = 8) with activity similar to that of VEGF165 but FGF2-induced cell migration was not impacted by EMCN knockdown compared to the control (1.05 ± 0.0433 vs. 1.22 ± 0.0491, *P* < 0.05, *n* = 8) (Fig. 5A). At the molecular level, FGF2-induced FGFR1 internalization after 45 min of stimulation (1.00 ± 0.0630 vs. 0.769 ± 0.0737, *P* < 0.05), and this internalization was not affected by EMCN knockdown compared to the control (1.03 ± 0.0905 vs. 0.734 ± 0.0746, *P* < 0.05, *n* = 7) (Fig. 5B).

We have previously shown, using immunoprecipitation, that EMCN associates with VEGFR2 [27, 33]. To investigate whether EMCN interacts with VEGFR1 and FGFR1, we utilized HRECs overexpressing Myc-tagged EMCN to facilitate the immunoprecipitation of EMCN as well as the identification of EMCN interacting proteins. Unlike VEGFR2, VEGFR1 and FGFR1 were not found to be associated with EMCN (*n* = 3) (Fig. 5C).

### EMCN knockdown impairs VEGF121-induced cell migration and VEGFR2 internalization

**Fig. 6:**
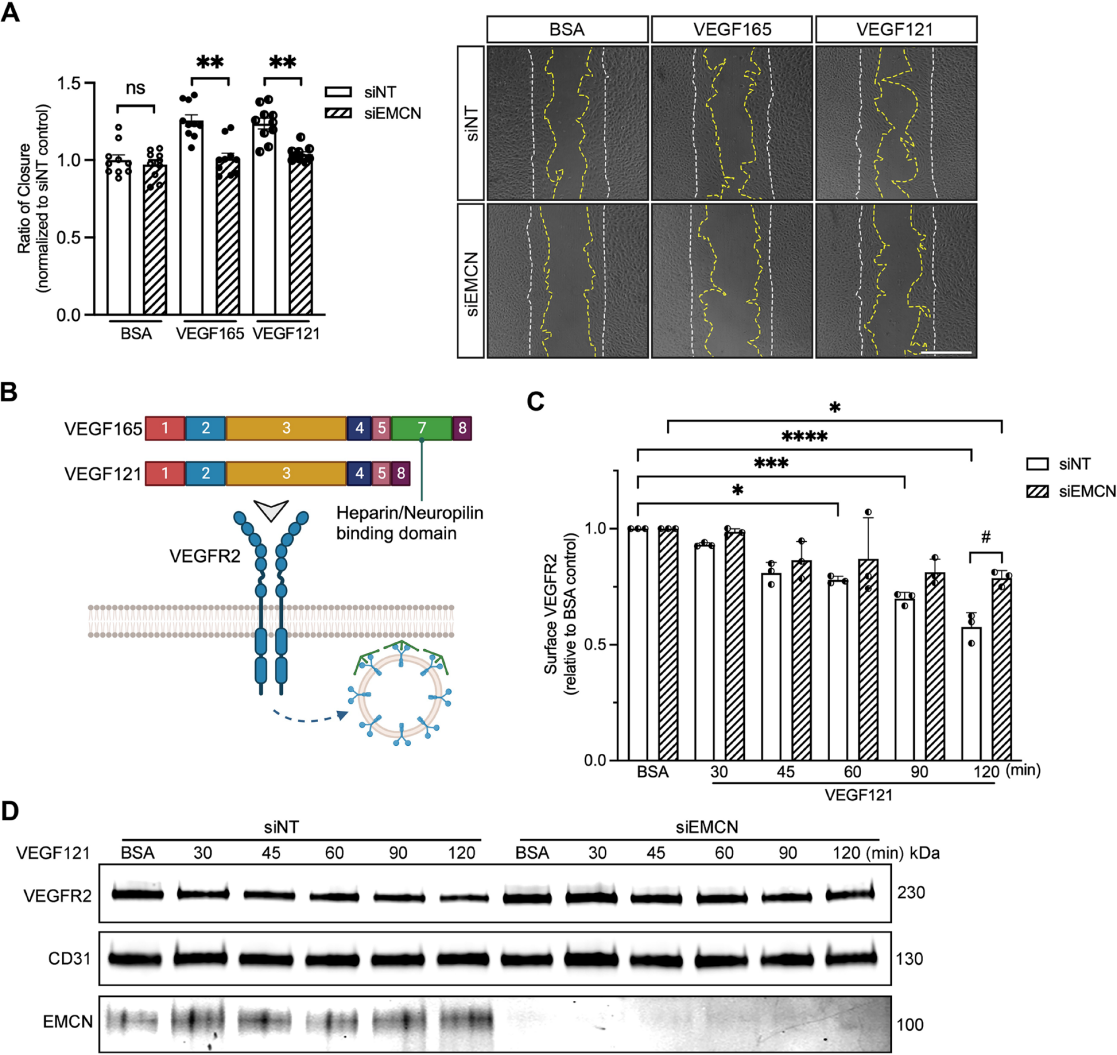
EMCN knockdown inhibits VEGF121 induced VEGFR2 internalization and HRECs migration similar to VEGF165. **A** Both VEGF165 (10 ng/ml)- and VEGF121 (7.29 ng/ml)-induced migration were inhibited with EMCN knockdown. Quantification of cell migration by image analysis is shown (left). Student-t test was used for comparisons between groups. *P < 0.05, **P < 0.01, *n* = 10. Representative images of each group at time zero (white dashed line) and 15 h time (yellow dashed line) points. The scale bar represents 500 µm. **B** Schematic representation of VEGFA isoforms, VEGF165 and VEGF121. **C** HRECs were treated with siEMCN stimulated with VEGF121 (7.29 ng/ml) for a time course of up to 120 min. VEGF121 induced significant VEGFR2 endocytosis after 60 min of stimulation, except when EMCN was knockdown. One-way ANOVA was used for comparation within group. Student-t test was used for comparation between siNT and siEMCN at the same time point. #*P* < 0.05, *P < 0.05, ***P < 0.001, ****P < 0.0001, *n* = 3. **D** Representative image of the western blot for VEGFR2 internalization in both siNT and siEMCN groups. CD31 was blotted as cell surface fraction loading control

VEGF165 and VEGF121, distinct VEGF isoforms produced by alternative mRNA splicing, differ in size and expression patterns as well as biochemical and biological properties [42, 47]. To determine if, like VEGF165, EMCN was necessary for VEGF121-induced VEGFR2 internalization, the effect of EMCN knockdown on VEGF121- and VEGF165- induced HREC migration was measured. Experiments were conducted at equimolar concentrations of 52 nM, which translates to a mass concentration of 7.29 ng/ml for VEGF121 and 10 ng/ml for VEGF165. VEGF121 significantly induced HREC migration compared to unstimulated controls (1.00 ± 0.0328 vs. 1.186 ± 0.0352, *P* < 0.001, *n* = 10). EMCN knockdown significantly reduced VEGF165- and VEGF121-induced HREC migration to a similar degree (1.15 ± 0.0235 vs. 1.01 ± 0.0348, *P* < 0.05 and 1.19 ± 0.0352 vs. 1.04 ± 0.0152, *P* < 0.01, respectively, *n* = 10) (Fig. 6A). The difference between VEGF165 and VEGF121 is represented in Fig. 6B. In line with the effects on HREC migration, VEGF121-induced significant VEGFR2 internalization after 60 min of stimulation (1.00 vs. 0.780 ± 0.00925, *P* < 0.05, *n* = 3). VEGFR2 internalization in HRECs continued up to 120 min following VEGF121 stimulation but was significant reduced in cells with EMCN knockdown (0.577 ± 0.0359 vs. 0.787 ± 0.0192, *P* < 0.05, *n* = 3) (Fig. 6C and D).

**Fig. 7:**
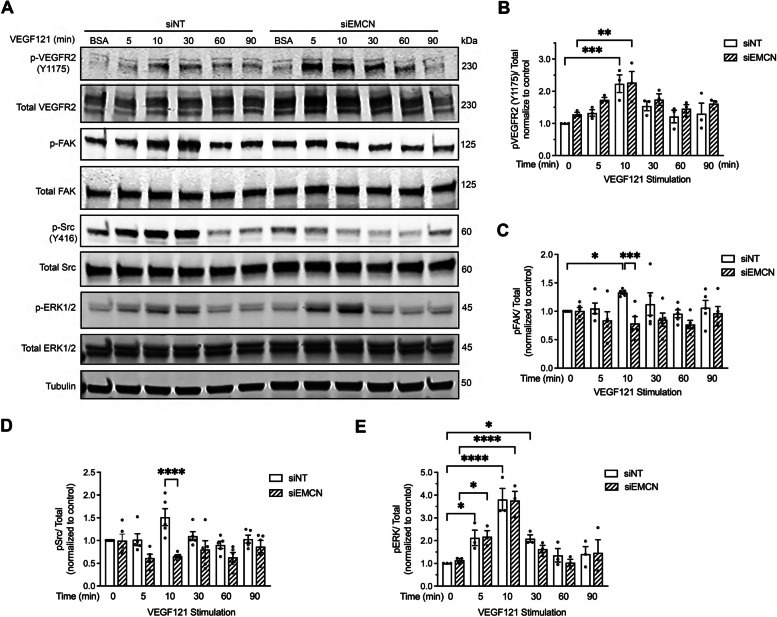
Depletion of EMCN reduces the phosphorylation of FAK and Src following VEGFR2 activation. **A** Western blot image of p-VEGFR2, total VEGFR2, p-Src, total Src, p-FAK, total FAK, p-ERK1/2, total ERK1/2, and tubulin probing for a VEGF121 stimulation time course with and without EMCN. An equal quantity of protein was loaded into each well. **B** Densitometric analysis of western blot data demonstrated that knockdown of EMCN did not alter VEGF121-induced VEGFR2 activation. *n* = 3. **C** Western blot analysis demonstrated that VEGF121 activation of FAK at 10 min. **D** and Src at 10 min was diminished **E** whereas ERK activation was not affected by EMCN knockdown. One-way ANOVA analysis was used for comparation between different time points within group. Student-t test was used for comparation between siNT and siEMCN at the same time point. Statistical analysis. *P < 0.05, **P < 0.01, ***P < 0.001, ****P < 0.0001 *n* ≥ 3

EMCN knockdown did not interfere with VEGF121-induced VEGFR2 phosphorylation (Fig. 7A and B), which is similar to our previous report for VEGF165 [33]. Upon phosphorylation, VEGFR2 signaling propagates to intracellular signaling molecules such as Src, FAK, and ERK1/2. Subsequent activation of Src at the Y416 site, FAK at the Y397 site, and ERK1/2 at the Y204/T202 sites has been shown to promote endothelial cell migration, proliferation, permeability, and survival [21, 22, 45]. VEGF121 induced peak activation of Src and FAK at around 10 min which was prevented upon EMCN depletion (1.516 $\pm$ 0.185 vs. 0.654 ± 0.0335, *P* < 0.001, *n* = 5 and 1.151 ± 0.120 vs. 0.786 ± 0.118, *P* < 0.05, *n* = 5) (Fig. 7A, C, and D). ERK signaling induced by VEGF121 was unperturbed by the knockdown of EMCN (Fig. 7E).

## Discussion

The findings presented here build upon previous work identifying EMCN as a regulator of VEGF signaling [44]. EMCN was demonstrated to promote VEGF-induced VEGFR2 endocytosis and, thus, downstream signaling and angiogenic activity in HRECs [33]. Truncation studies of the extracellular domain of EMCN revealed that the first 21–121 amino acids are required for VEGFR2 interaction and internalization. Furthermore, N-glycosylation of the extracellular domain of EMCN was also determined to be required for interaction with VEGFR2 [27]. In this study, we investigated the molecular mechanism by which EMCN mediates the activity of VEGFR2 as well as the specificity of EMCN for the different tyrosine kinase receptors.

VEGFR2 plays a critical role in VEGF signaling and is essential for orchestrating numerous cellular responses required for angiogenesis, such as vascular permeability, endothelial migration, proliferation, and tube formation. CME has a crucial function in regulating the endocytosis of cell surface receptors, which begins with the creation of pits on the inner surface of the cytoplasmic membrane, including clathrin, the AP2 adaptor protein complex, and accompanying proteins [14]. Our data indicated a group of potential EMCN-binding proteins identified by mass spectrometry analysis that are involved in CME, highlighting the crucial role of EMCN in regulating VEGF-induced VEGFR2 internalization via CME [26, 33]. AP2 is a core adaptor in CME that is one of the first proteins to arrive at a forming clathrin-coated vesicle and is crucial for initiating clathrin polymerization [8, 29]. Since clathrin cannot directly interact with the lipids or proteins of the plasma membrane [37], adaptor proteins assist in the assembly of clathrin-coated vesicles by providing a link between clathrin and the membrane-bound cargo. Our data further supports this finding, as knockdown of the AP2A2 subunit impeded VEGFR2 endocytosis. We found that the absence of EMCN resulted in a reduction in the colocalization of the clathrin heavy chain with VEGFR2, but not in the colocalization of clathrin heavy chain with AP2. The AP2 complex plays a crucial role in facilitating the recruitment of clathrin to the plasma membrane at sites where cargo molecules, such as VEGFR2, are destined for internalization through the recognition and binding of specific motifs [30]. We demonstrated that EMCN interacts with AP2A2 subunit and here we further showed that EMCN interacts with the AP2 complex as evident by co-immunoprecipitation of both the α and β subunits with EMCN. Knocking down EMCN resulted in reduced interaction between VEGFR2 and the AP2A2 subunit compared to the control condition. Our data provide new insight into the molecular mechanism by which EMCN is required for VEGFR2 interaction with the AP2 complex, which in turn recruits clathrin to the endocytic pits.

Like VEGFR2, VEGFR1 and FGFR1 undergo ligand-induced CME as a critical component of their signaling [21, 53]. We have shown that EMCN is necessary for VEGF-induced VEGFR2 CME [33], so we explored whether VEGFR1 and FGFR1 require interacting with EMCN for CME. The functional and biochemical data indicate that EMCN’s role is specific for VEGFR2 in ECs. Neither PlGF-2 nor FGF2-induced endothelial migration nor VEGFR1 and FGFR1 receptor internalization required the presence of EMCN. The finding that VEGF165-induced VEGFR1 endocytosis was not impacted by EMCN depletion suggests that the mechanisms of VEGFR1 CME is distinct from that for VEGFR2. Following ligand stimulation, both VEGFR1 and FGFR1 undergo CME, independent of EMCN, to induce signaling. In the absence of EMCN, only ligand-induced VEGFR2 CME and subsequent angiogenesis signaling was prevented. The interaction of VEGFR2 with EMCN is necessary for VEGFR2 to associate with adaptor proteins in the CME complex and the interaction appears to be transient as EMCN is not internalized with VEGFR2 [33]. To understand how EMCN is involved in VEGFR2 CME but not VEGFR1 or FGFR1, we assessed the interaction between EMCN and these receptors. Unlike the interaction observed with VEGFR2, we found that EMCN does not interact with VEGFR1 and FGFR1. This finding supports the selectivity of EMCN's involvement in VEGFR2 CME and provides insights into the mechanistic differences governing receptor internalization processes.

VEGFR2 activity is contingent on the presence and interaction with ligands, adaptor proteins, and co-receptors [1, 51, 61]. While VEGF165 and VEGF121 both induce angiogenesis through VEGFR2 activation, only VEGF165 induces an inflammatory response, including the upregulation of adhesion molecules by ECs [36, 57]. VEGF165 and VEGF121 differ by the presence and absence of the exon 7 encoded heparin/neuropilin binding domain, respectively [6, 41, 47]. While angiogenesis and inflammatory signaling are attributed to VEGF165 [28], VEGF121 has only been reported to induce angiogenic signaling. VEGF121, like VEGF165, activates VEGFR2 at the Y1175 residue with a peak at about 10 min, which produces an angiogenic signal [33]. Neuropilin-1 (NRP1) is a well-studied co-receptor for VEGFR2 that plays a significant role in VEGF165 signaling. Interfering between NRP1 and VEGF165 binding prevents the receptor complex from downstream signaling, while VEGF121-induced VEGFR2 signaling is unperturbed [59]. Our previous findings demonstrate that EMCN associates with VEGFR2 and that this interaction is necessary for VEGF165-induced VEGFR2 internalization [27]**.** Under the conditions employed for VEGF165, VEGF121-induced VEGFR2 internalization was not observed, and thus a time course was conducted. We established that VEGF121-induction of VEGFR2 internalization begins at about 60 min and continues until at least 120 min and that EMCN depletion reduced VEGF121-induced HREC migration and prevented VEGFR2 internalization similar to that seen for VEGF165. Additionally, VEGF121-induced phosphorylation of VEGFR2 at the Y1175 site was consistent with our previous report investigating VEGF165 activation of VEGFR2 after the knockdown of EMCN [33]. These results showed that VEGFR2 phosphorylation upon ligand binding does not require the presence of EMCN,however, EMCN, due to its role in receptor internalization, is essential for modulating downstream signaling that leads to VEGF-induced biological activity.

VEGF165-induced VEGFR2 activity has been clearly shown to be critical for developmental angiogenesis and several pathologies [28], such as in neovascular age-related macular degeneration and tumor growth. That said, VEGFR2 expression and functions are not specific to the endothelium. VEGFR2 is also expressed by hematopoietic cells, neuronal cells, osteoblasts, pancreatic duct cells, retinal progenitor cells, and megakaryocytes [38] and is involved in neurodevelopment and neuroprotection, monocyte/macrophage recruitment, maintenance of barrier functions, and promoting cell survival [19, 48, 49]. The findings reported here suggest a specific role for EMCN in modulating ligand-driven VEGFR2 CME and signaling. While VEGF signaling has been targeted clinically both by neutralizing the ligand and blocking the receptor, neither of those approaches is endothelial-specific and VEGF-signaling has been shown to be involved in a variety of non-endothelial functions, thus leading to “off-target” effects. The ability to block VEGF signaling by targeting the interaction of EMCN, an endothelial-specific molecule, with VEGFR2 with a blocking antibody, would provide a level of specificity that is currently not available.

### Experimental procedures

#### Cell culture

Primary human retinal EC (HRECs) at passage (P) 3 were purchased from Cell Systems (ACBRI 181). HRECs were cultured at 37 °C and in 5% CO_2_. Endothelial Basal Media-2 (EBM-2) BulletKit Medium (Lonza, # CC-3162) supplemented with 2% fetal bovine serum (FBS, Atlanta Biologicals) and 2 mM L-glutamine (Lonza, # CC-17-605E) was used to maintain HRECs. Culture plates were coated in 0.2% gelatin from porcine skin (Sigma-Aldrich, #G1890) for 30 min at 37˚C. HRECs establish a monolayer with 95–100% confluence on the day of the experiment. Cells were used up to P9.

#### Reagents

Recombinant human VEGF165 (#293-VE), PlGF-2 (#6837-PL), and FGF2 (#233-FB) were purchased from R&D Systems. VEGF121 (#8908) was obtained from Cell Signaling. Phosphatase inhibitor cocktail tablet (#4906845001), protease inhibitor cocktail table (#5892970001), primaquine bisphosphate (PQB, # 160393-1G), phosphate-buffered saline (PBS, #D5652-10 × 1L), DL-Dithiothreitol (DTT, # D9163-5G), bovine serum albumin (BSA, #A6003), and Tween-20 (#X251-07) were from Sigma-Aldrich. Sulfo-NHSS-SS-Biotin (#1859385) and avidin agarose resin (#S1258122) were from Life Technologies. Protein A/G beads (#sc-2003) and mouse IgG (#sc-2025) were from Santa Cruz. Cell lysis buffer (# 9803S) was from Cell Signaling. Tris-buffered saline (TBS, #170–6435), Mini-PROTEAN TGX gels (#4561094), Tris/Glycine/SDS Buffer (#1610772), Tris/Glycine Buffer (#1610771), and Precision Plus Protein Dual Color Standards (#161–0374), were obtained from Bio-Rad. BioTrace™ NT Nitrocellulose Transfer Membrane 30 cm × 3 m roll (#27376–991) was purchased from Fisher Scientific.

### Antibodies

Immunoblots were probed with rabbit anti-p-VEGFR2-Y1175 (1:1,000, Cell Signaling, #2478S), rabbit anti-Src (1:1,000, Cell Signaling, #2109S), rabbit anti-pSrc-Ser17 (1:1,000, Cell Signaling, #12432), rabbit anti-FAK (1:1,000, Novus Biologicals, #NBP2-67327), rabbit anti-p-FAK-Y397 (1:1,000, Abcam, #ab81298), rabbit anti-p-ERK1/2 Y204/ T202 (1:1,000, Cell Signaling, #4370S), mouse anti-ERK1/2 (1:1,000, Cell Signaling, #4696S), rabbit anti-VEGFR2 (1:1,000, Cell Signaling, #2479S), goat anti-VEGFR1 (1:1000, R&D Systems #AF321), rabbit anti-FGFR1 (1:1000, Cell Signaling, #9740S), mouse anti-Myc (1:1000, Cell Signaling, #9B11) and mouse anti-CD31 (1:1,000, Thermo Fisher, #14–0311-81). Secondary antibodies used include goat anti-rabbit 800CW (1:20,000, Li-cor, #925–32211), goat anti-mouse 680RD (1:20,000, Li-cor, #925–68070), donkey anti-goat 800CW (1:20,000, Li-cor, #926–32214) goat anti-rabbit horseradish peroxidase (HRP) conjugated (1:1000, R&D Systems #HAF008), goat anti-mouse HRP conjugated (1:1000, R&D Systems #HAF007), and donkey anti-goat HRP conjugated (1:1000, R&D Systems #HAF109).

### Identifying EMCN binding partners by mass spectrometry

Confluent HRECs were collected and centrifuged at 1,500 rcf for 5 min at 4 °C. The supernatant was discarded, and the pellet was suspended and then incubated with lysis buffer with proteinase inhibitors for 30 min on ice with gentle mixing. The lysate was centrifuged at 16,000 rcf at 4 °C. Twenty percent of the supernatant was saved as the sample input. The remaining supernatant was incubated with rabbit anti-EMCN antibody (1:50, Invitrogen, #712677) or mouse IgG rotating overnight at 4 °C. Protein A/G beads were washed with 0.2% PBST and then blocked in 3% BSA, 0.1% PBST at 4 °C overnight. The following day, the beads were washed in PBS and incubated with lysate at 4 °C overnight. After three rinses with 0.1% PBST, bound EMCN-binding proteins were eluted using Laemmli’s SDS sample buffer with 100 mM DTT and boiled at 95 °C for 10 min. Immunoprecipitated EMCN-binding proteins were separated on 5% SDS-PAGE gel for 30 min at 60 V. The gel was then washed with PBS and incubated overnight with Coomassie Blue and followed by destaining using PBS. The EMCN-binding proteins were excised together with the IgG control lane and sent to Harvard Medical School Taplin Mass Spectrometry Core Facility for liquid chromatography with tandem mass spectrometry analysis. The annotated EMCN-specific binding proteins were further analyzed using STRING database for further functional clustering.

### siRNA knockdown

Non-targeting siRNA (siNT) (50 nM, Dharmacon #D-001810–01-20) or siEMCN (50 nM, Dharmacon #L-051510–01-0005) was incubated for 30 min at room temperature with DharmaFECT transfection reagent (Dharmacon, # T-2001–03) in Opti-MEM™ (Life Technologies, # 51985034). The siRNA complex was added to the HRECs in complete EBM-2 media without penicillin–streptomycin overnight and removed with a change of medium. The efficiency of the EMCN knockdown was described previously by our lab [43] and in this paper (Supplementary Fig. 2). For HREC migration and receptor internalization experiments, HRECs were treated with siRNA 48 h prior to experimentation.

### Adenovirus overexpression

HRECs were seeded at a confluency of 70% 24 h prior to transduction with an adenovirus expressing myc-tagged human EMCN (AdEMCN-myc). The adenovirus was added at a multiplicity of infection of 30 in EBM-2 media supplemented with 2% FBS.

### ICC-based internalization assay

HRECs were serum starved for 2 h in EBM-2 and incubated with goat anti-VEGFR2 (1:100, AF357; R&D Systems) at 4 °C for 1 h, followed by the addition of bovine serum albumin (BSA) or VEGF (10 ng/ml) for 30 min at 37 °C. Cells were fixed in 4% PFA for 5 min at room temperature and permeabilized using 0.1% Triton X-100. At this time, cells were incubated with rabbit anti-clathrin (1:200, 4796 T; Cell Signaling Technology) to visualize colocalization between VEGFR2 and clathrin. All cells were then incubated with Alexa Fluor 594–labeled donkey anti-goat (1:300, A-11058) and Alexa Fluor 647–labeled donkey anti-rabbit (1:300, A-31573). All experiments were conducted in the presence of PQB (0.6 μM) to prevent receptor recycling. Five images per cover slip were imaged using an Axioscop 2 Mot Plus microscope (Carl Zeiss, Oberkochen, Germany; 40 × magnification), analyzed using Photoshop 2023 (Adobe, San Jose, CA, USA), and averaged. Intracellular fluorescence intensity was quantified using Photoshop 2023 and normalized to the total number of cells per viewing field as an indication of receptor internalization. Quantification represents three independent experiments.

### Colocalization assay

HRECs were transfected with siRNA targeting at EMCN or non-targeting control and seeded on 24-well plates. HRECs were then serum starved for 2 h in basal EBM-2 media and stimulated with 10 ng/ml VEGF165 or 10 ng/ml BSA as control for 5 min before washing and fixation with 4%PFA. After 4% PFA fixation at room temperature and permeabilization using 5% serum in PBS with 0.1% Triton X-100, cell was then incubated with goat anti-VEGFR2 (1:200, AF357; R&D Systems), Rabbit anti clathrin (1:200, 4796 T; Cell Signaling Technology) and mouse anti AP2 (1:200, F-12, Santa Cruz) at 4 °C for overnight. All cells were then washed and incubated with Alexa Fluor 488-labeled donkey anti-rabbit (1:300, A-21206), Alexa Fluor 594-labeled donkey anti-mouse (1:300, A-21203) or Alexa Fluor 594–labeled donkey anti-goat (1:300, A-11058). Six to eight images per cover slip were imaged using SP8 Confocal Microscope (Leica, Germany; 80 × magnification), analyzed using Image J (Adobe, San Jose, CA, USA) JaCoP plugin. Quantification represents three independent experiments and eighteen images were analyzed for each condition.

### Co-immunoprecipitation

HRECs were plated onto 150 mm dishes and cultured in complete EBM-2 media. Cells were transduced with AdEMCN-myc and cultured for up to 72 h until confluent. The HRECs were collected in SFM and centrifuged at 1,500 rcf for 5 min at 4 °C. The supernatant was discarded, and the pellet was suspended and then incubated with lysis buffer with proteinase inhibitors for 30 min on ice with some gentle mixing. The lysate was centrifuged at 16,000 rcf at 4 °C. Twenty percent of the supernatant was saved as the input sample. The remaining supernatant was divided equally and incubated with either mouse anti-Myc antibody or mouse IgG at a 1:25 ratio, rotating overnight at 4 °C. Protein A/G beads were prepared by partitioning 70 µl of slurry per sample and washed with 0.2% PBST. The beads were centrifuged at 3,000 rcf for 5 min at 4 °C. The beads were then blocked in 3% BSA, 0.5% PBST at 4 °C overnight. The following day, the beads were washed in PBS and incubated with lysate overnight. The lysate was then washed from the beads with 0.2% PBST washes followed by PBS washes. Bound proteins were eluted and prepared for western blot processing using Laemmli’s SDS sample buffer with 100 mM DTT and incubation at 95 °C for 10 min.

### Western blot

Protein samples were eluted from the beads and loaded in 4–20% Mini-PROTEAN TGX gels in Tris–glycine SDS buffer and transferred onto a nitrocellulose transfer paper in Tris–glycine buffer. Membranes were blocked in 3% BSA in PBS and incubated with primary antibodies overnight. Membranes were then washed in PBST and incubated with secondary antibodies for 1 h. Images were developed using SuperSignal™ West Pico Chemiluminescent Substrate (Thermo Scientific, #34077) or fluorescence LI-COR Odyssey. ImageJ software was used for densitometric analysis of protein fluorescence [23]. Loading controls and total proteins were included for normalization, and data were presented as a fold change.

### Cell surface receptor internalization

Confluent HRECs were incubated in SFM for 2 h. The HRECs were then stimulated with indicated growth factors for 30–120 min in the presence of PQB. HRECS were incubated with sulfo-NHSS-SS-biotin in PBS at 4 °C for 30 min. The reaction was quenched with 50 mM Tris (pH 8.0), followed by PBS (pH 8.0). Cells were collected, lysed, sonicated in cell lysis buffer, and the lysate was collected at 10,000 rcf for 5 min. The supernatant was incubated with 100 µl of avidin agarose resin for 1 h at room temperature while rotating. The beads were spun down at 1,000 rcf and the supernatant was removed. The beads were washed three times using a 20 mM Tris–HCl, pH 6.8, 0.5% Tween-20 buffer. Laemmli's SDS Sample Buffer with 100 mM DTT was added to the beads. The beads were incubated at 95 °C for 10 min and prepared for western blot analysis. The protocol is illustrated in Fig. 4B.

### Endothelial cell migration

Confluent HRECs were incubated in serum-free basal EBM-2 media for 8 h. A p200 pipette tip was used to create a scratch across the monolayer of HRECs. The media were changed to serum-free basal EBM-2 media (SFM) with treatment groups. Images of the scratch wound areas were taken immediately post-treatment (time zero) and again after 15 h of migration. The latest version of the ImageJ MRI Wound Healing Tool plugin was used to analyze the migration area of the HRECs.

### Statistical analysis

Data are presented as the mean with a standard error of at least three independent experiments. “N” represents biological replicates. Statistical significance was determined using 2-Tailed Unpaired Student’s t-test, One-way ANOVA, or Two-way ANOVA (Prism 9 software, GraphPad, La Jolla, CA). Šidák posthoc testing was conducted on ANOVA analyses. Values of *P* < 0.05 were considered statistically significant.

### Supplementary Information

**Additional file 1:** **Supplemental Figure 1**. Markov Clustering of EMCN-binding proteins identified by mass spectrometry. (A) EMCN-binding proteins identified through mass spectrometry were analyzed and visualized using the STRING database (https://string-db.org/). The network type was set to physical subnetwork, and the significance of network edges was based on confidence, with a minimum required interaction score of high confidence (0.700). Further functional clustering of EMCN-binding proteins was performed using Markov clustering. Proteins within the same cluster were connected by solid lines and marked with the same color, while dashed lines indicated boundaries between clusters. (B) The table lists the top five protein clusters with the highest number of proteins. **Supplemental Figure 2.** Confirmation of EMCN knockdown. (A) Representative image of the western blot assay showing significant reduction of EMCN at protein level in HRECs transfected with siEMCN compared to siNT control. (B) Quantification of EMCN protein by western blot analysis demonstrates a significant decrease of EMCN at 72 hr after transfection. Student-t test was used. ****P<0.0001, *n*=3. (C) Quantification of EMCN mRNA indicates a significant decrease of EMCN (1.024 ± 0.126 vs. 0.0128 ± 0.0003) at 48 hr after transfection. Student-t test was used. ****P<0.0001, *n*=3.

## Data Availability

All data described are contained within the manuscript.

## Abbreviations

EMCN: Endomucin
VEGF: Vascular endothelial growth factor
VEGFR2: Vascular endothelial growth factor receptor 2
VEGFR1: Vascular endothelial growth factor receptor 1
FGF2: Basic fibroblast growth factor
FGFR1: Fibroblast growth factor receptor 1
HRECs: Human retinal endothelial cells
CME: Clathrin-mediated endocytosis
NRP1: Neuropilin-1
PIGF: Placental growth factor
PQB: Primaquine bisphosphate
