# Correction: Endomucin selectively regulates vascular endothelial growth factor receptor-2 endocytosis through its interaction with AP2

**DOI:** 10.1186/s12964-024-01696-6

**Published:** 2024-06-06

**Authors:** Issahy Cano, Melissa Wild, Urvi Gupta, Suman Chaudhary, Yin Shan Eric Ng, Magali Saint-Geniez, Patricia A. D’Amore, Zhengping Hu

**Affiliations:** 1 Schepens Eye Research Institute of Massachusetts Eye and Ear, Boston, MA, USA; 2 Department of Ophthalmology, Harvard Medical School, Boston, MA, USA; 3 Department of Pathology, Harvard Medical School, Boston, MA, USA; 4 Case Western Reserve University School of Medicine, Cleveland, OH, USA; 5 Present Address: Department of Molecular Medicine, Cornell University, Ithaca, NY, USA; 6 Present Address: EyeBiotech, London, UK; 7 Present Address: Novartis Institutes for Biomedical Research, Cambridge, MA, USA

**Correction: Cell Commun Signal 22, 225 (2024)**

10.1186/s12964-024-01606-w

Following publication of the original article [1], the authors reported an error in the additional file. The incorrect additional file was published. The correct file is published in this correction article and the original article [1] has been corrected.

### Electronic supplementary material

Below is the link to the electronic supplementary material.

Additional file 1: **Supplemental Fig. 1**. Markov Clustering of EMCN-binding proteins identified by mass spectrometry. (A) EMCN-binding proteins identified through mass spectrometry were analyzed and visualized using the STRING database (https://string-db.org/). The network type was set to physical subnetwork, and the significance of network edges was based on confidence, with a minimum required interaction score of high confidence (0.700). Further functional clustering of EMCN-binding proteins was performed using Markov clustering. Proteins within the same cluster were connected by solid lines and marked with the same color, while dashed lines indicated boundaries between clusters. (B) The table lists the top five protein clusters with the highest number of proteins. **Supplemental Fig. 2.** Confirmation of EMCN knockdown. (A) Representative image of the western blot assay showing significant reduction of EMCN at protein level in HRECs transfected with siEMCN compared to siNT control. (B) Quantification of EMCN protein by western blot analysis demonstrates a significant decrease of EMCN at 72 h after transfection. Student-t test was used. ****P < 0.0001, *n* = 3. (C) Quantification of EMCN mRNA indicates a significant decrease of EMCN (1.024 ± 0.126 vs. 0.0128 ± 0.0003) at 48 h after transfection. Student-t test was used. ****P < 0.0001, *n* = 3.

## References

[1] Cano I, Wild M, Gupta U, et al. Endomucin selectively regulates vascular endothelial growth factor receptor-2 endocytosis through its interaction with AP2. Cell Commun Signal. 2024;22:225. 10.1186/s12964-024-01606-w. 10.1186/s12964-024-01606-w PMC11007909 38605348

