# Supplementary material for: Correction: Endomucin selectively regulates vascular endothelial growth factor receptor-2 endocytosis through its interaction with AP2
Source: Cell Commun Signal. 2024 Jun 6;22:311. doi: 10.1186/s12964-024-01696-6 (PMC11155063; doi:10.1186/s12964-024-01696-6)

Supplementary Figure 1

A

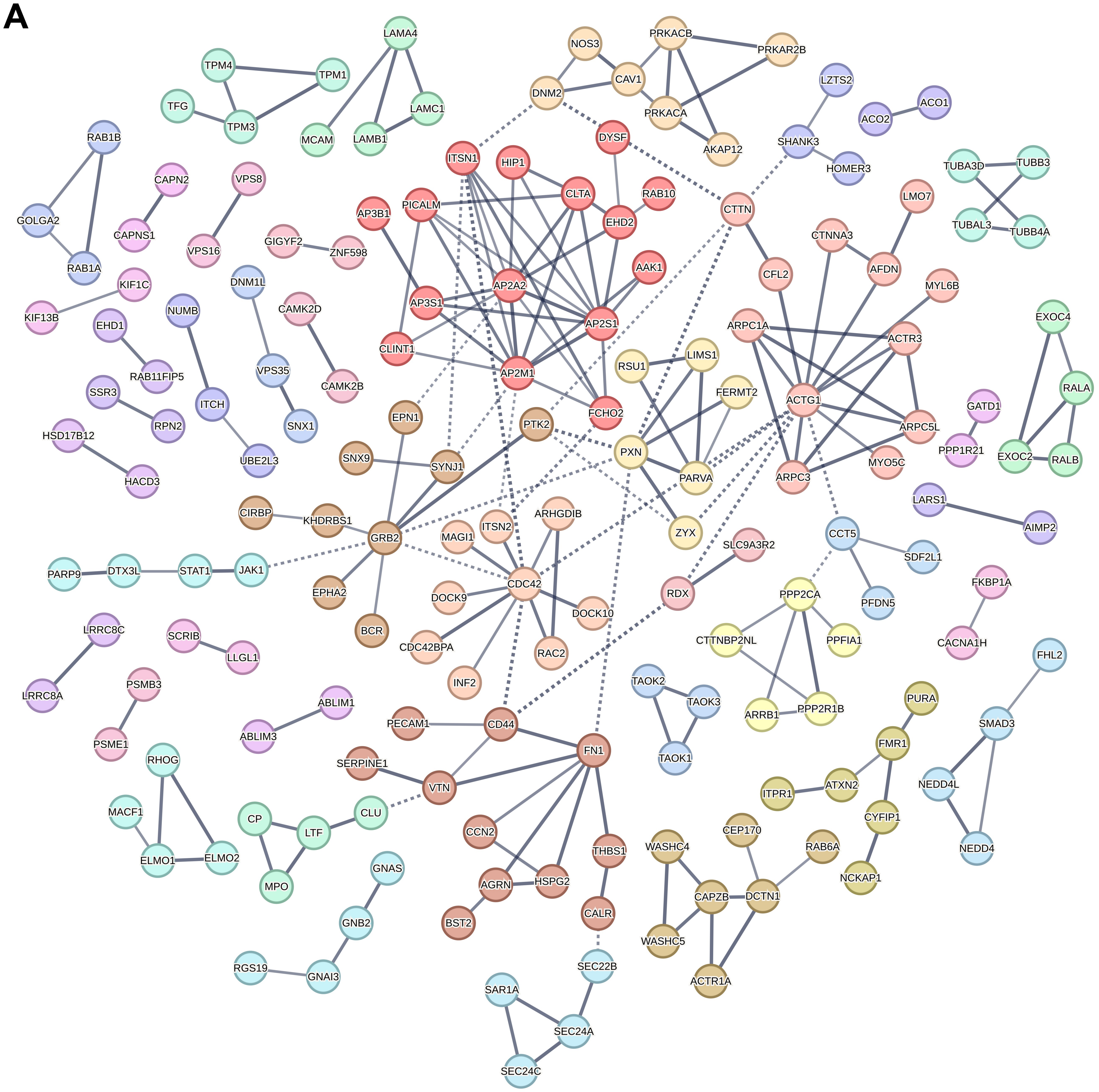

B

Top gene clusters

bubble	gene count	gene ontology	protein names
●	15	clathrin coat, endocytosis	AAK1,AP2A2,AP2M1,AP2S1,AP3B1,AP3S1,CLINT1,CLTA,DYSF,EHD2,FCHO2 HIP1,ITSN,PICALM,RAB10
●	12	actin filament polymerization and organization	ACTG1,ACTR3,AFDN,ARPC1A,ARPC3,ARPC5L,CFL2,CTNNA3,CTTN,LMO7 MYL6B,MYO5C
●	11	extracellular matrix organization	AGRN,BST2,CALR,CCN2,CD44,FN1,HSPG2,PECAM1,SERPINE1,THBS1,VTN
●	9	small GTPase mediated signal transduction	ARHGDIB,CDC42,CDC42BPA,DOCK10,DOCK9,INF2,ITSN2,MAGI1,RAC2
●	9	signal transduction	BCR,CIRBP,EPHA2,EPN1,GRB2,KHDRBS1,PTK2,SNX9,SYNJ1

Supplementary Figure 2

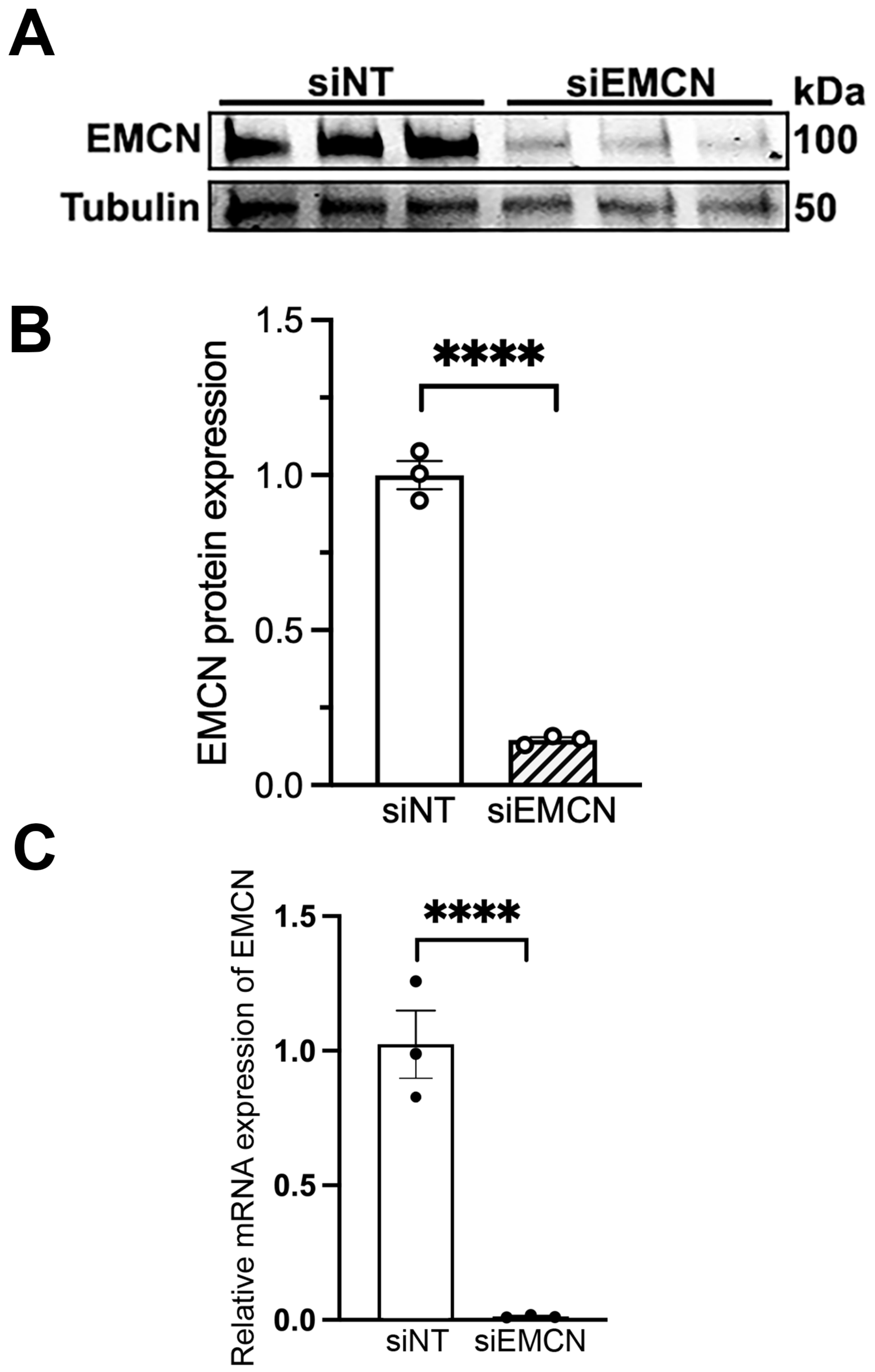

Supplement: Supplementary file 1 — Additional file 1: Supplemental Fig. 1. Markov Clustering of EMCN-binding proteins identified by mass spectrometry. (A) EMCN-binding proteins identified through mass spectrometry were analyzed and visualized using the STRING database (https://string-db.org/). The network type was set to physical subnetwork, and the significance of network edges was based on confidence, with a minimum required interaction score of high confidence (0.700). Further functional clustering of EMCN-binding proteins was performed using Markov clustering. Proteins within the same cluster were connected by solid lines and marked with the same color, while dashed lines indicated boundaries between clusters. (B) The table lists the top five protein clusters with the highest number of proteins. Supplemental Fig. 2. Confirmation of EMCN knockdown. (A) Representative image of the western blot assay showing significant reduction of EMCN at protein level in HRECs transfected with siEMCN compared to siNT control. (B) Quantification of EMCN protein by western blot analysis demonstrates a significant decrease of EMCN at 72 h after transfection. Student-t test was used. ****P < 0.0001, n = 3. (C) Quantification of EMCN mRNA indicates a significant decrease of EMCN (1.024 ± 0.126 vs. 0.0128 ± 0.0003) at 48 h after transfection. Student-t test was used. ****P < 0.0001, n = 3. [file 12964_2024_1696_MOESM1_ESM.pdf]
